# Erratum for “Effect of phenylbutazone on insulin secretion in horses with insulin dysregulation”

**DOI:** 10.1111/jvim.17210

**Published:** 2024-10-07

Kemp KL, Skinner JE, Bertin FR. Effect of phenylbutazone on insulin secretion in horses with insulin dysregulation. *J Vet Intern Med*. 2024;38(2):1177‐1184. doi:10.1111/jvim.17013

The phenylbutazone solution used in the study contained 200 mg/mL of phenylbutazone sodium and 50 mg/mL of sodium salicylate.

